# Effects of Urban Green Space on Cardiovascular and Respiratory Biomarkers in Chinese Adults: Panel Study Using Digital Tracking Devices

**DOI:** 10.2196/31316

**Published:** 2021-12-30

**Authors:** Lin Yang, Ka Long Chan, John W M Yuen, Frances K Y Wong, Lefei Han, Hung Chak Ho, Katherine K P Chang, Yuen Shan Ho, Judy Yuen-Man Siu, Linwei Tian, Man Sing Wong

**Affiliations:** 1 School of Nursing The Hong Kong Polytechnic University Hong Kong Hong Kong; 2 Department of Land Surveying and Geo-Informatics The Hong Kong Polytechnic University Hong Kong Hong Kong; 3 School of Global Health Chinese Center for Tropical Diseases Research Shanghai Jiao Tong University School of Medicine Shanghai China; 4 Department of Urban Planning and Design The University of Hong Kong Hong Kong Hong Kong; 5 Department of Applied Social Sciences The Hong Kong Polytechnic University Hong Kong Hong Kong; 6 School of Public Health The University of Hong Kong Hong Kong Hong Kong

**Keywords:** green space, biomarker, cardiovascular disease, respiratory disease

## Abstract

**Background:**

The health benefits of urban green space have been widely reported in the literature; however, the biological mechanisms remain unexplored, and a causal relationship cannot be established between green space exposure and cardiorespiratory health.

**Objective:**

Our aim was to conduct a panel study using personal tracking devices to continuously collect individual exposure data from healthy Chinese adults aged 50 to 64 years living in Hong Kong.

**Methods:**

A panel of cardiorespiratory biomarkers was tested each week for a period of 5 consecutive weeks. Data on weekly exposure to green space, air pollution, and the physical activities of individual participants were collected by personal tracking devices. The effects of green space exposure measured by the normalized difference vegetation index (NDVI) at buffer zones of 100, 250, and 500 meters on a panel of cardiorespiratory biomarkers were estimated by a generalized linear mixed-effects model, with adjustment for confounding variables of sociodemographic characteristics, exposure to air pollutants and noise, exercise, and nutrient intake.

**Results:**

A total of 39 participants (mean age 56.4 years, range 50-63 years) were recruited and followed up for 5 consecutive weeks. After adjustment for sex, income, occupation, physical activities, dietary intake, noise, and air pollution, significant negative associations with the NDVI for the 250-meter buffer zone were found in total cholesterol (–21.6% per IQR increase in NDVI, 95% CI –32.7% to –10.6%), low-density lipoprotein (–14.9%, 95% CI –23.4% to –6.4%), glucose (–11.2%, 95% CI –21.9% to –0.5%), and high-sensitivity C-reactive protein (–41.3%, 95% CI –81.7% to –0.9%). Similar effect estimates were found for the 100-meter and 250-meter buffer zones. After adjustment for multiple testing, the effect estimates of glucose and high-sensitivity C-reactive protein were no longer significant.

**Conclusions:**

The health benefits of green space can be found in some metabolic and inflammatory biomarkers. Further studies are warranted to establish the causal relationship between green space and cardiorespiratory health.

## Introduction

### Background

Previous studies have demonstrated the health benefits of the natural environment and urban green space on mental health [1,2], perceived stress [3-5], sleep quality [6,7], and cardiovascular and respiratory health [8]. The health benefits of green space in neighborhoods may be due to increased physical activity, reduced air pollution exposure, and relief of stress from work and life [9-12]. A prospective cohort study in the United States also showed that people living in communities with higher green space coverage had a lower mortality rate, and this association was likely mediated by physical activity and air pollution [13]. However, to date, the underlying mechanism remains unexplored in the literature, which hinders the establishment of a causal relationship between green space exposure and cardiorespiratory health. In environmental health studies, the panel study design has often been adopted to investigate the short-term impacts of environmental factors on cardiovascular and respiratory health by comparing the levels of metabolic, inflammatory, and oxidative biomarkers of the same cohort of volunteers at different time points [14-16]. The panel study design features repeated collection of samples from the same individuals at different times, with the aim to demonstrate the changes in biomarkers at various exposure levels. As each individual serves as their own control, this study design minimizes the confounding of time-invariant factors (such as demographics and health-seeking behavior) but is subject to the confounding of other temporal factors, such as air pollution [17]. In addition, a panel study requires good-quality personal exposure data, which are often absent in many regions. Fortunately, this obstacle has been diminished by the recent research development of combining personal tracking devices with satellite images to estimate individual exposures to air pollution and actual access to green space [18].

### Objective

With the aim to explore the effects of green space on respiratory and cardiovascular biomarkers to provide evidence for the underlying biological pathways, we conducted a panel study using personal tracking devices to continuously collect the individual exposure data in healthy Chinese adults aged 50 to 64 years living in Hong Kong. Using these data, we estimated the independent effects of green space exposure on different metabolic, inflammatory, and oxidative biomarkers for cardiovascular and respiratory health.

## Methods

### Study Design and Participants

The target population was Chinese adults aged 50 to 64 years who had been living in Hong Kong for the past 2 years. This age group was chosen because of their high risk of preclinical chronic conditions [19]. Inclusion criteria were nonsmoker or no exposure to secondhand smoke at home or work; generally healthy without any diagnosed chronic diseases or regular taking of medicines; no need of walking assistance; and staying at the same residential address during the whole study period. We recruited participants using convenience sampling via posters and social media and through snowball sampling by inviting participants to refer their friends to us.

Using the formula for mixed models with repeated measurements [20], we calculated the sample based on the results from one previous study on indoor and outdoor air pollution [21], which used a similar study design. We assumed that the mean concentration of 8-hydroxy-2'-deoxyguanosine (8-OHdG) measurements was 4.0 ng/mL, with a standard deviation of 2.3. The sample size of 30 could achieve 90% power to detect an effect size of 0.8, under the assumption that the autocorrelation of repeated measurements was 0.9. Given the 20% attrition rate during the repeated measurements, we decided to recruit 38 to 40 participants for this study.

The participants were recruited and followed up for 5 consecutive weeks during October to December 2017. On the same weekday of each week during the study period, each individual participant was invited to visit the Integrative Health Clinic (IHC) of the Hong Kong Polytechnic University for blood sample collection and physical examination. Each participant had a total of 6 visits during the whole study period (1 at enrollment and 5 at follow-up). These visits were scheduled on early mornings (8 AM-10 AM) of the same weekday to reduce the bias caused by the diurnal change of biomarkers. If participants took any medication, experienced allergies in the preceding 7 days, or worked a night shift on the day before their scheduled visit, these visits would be postponed for 1 week.

A summary of the data collection procedure at the clinic visits is shown in Table S1 in Multimedia Appendix 1. At recruitment (the first visit to the IHC), each participant signed a consent form and took a self-administered questionnaire, with the support of research assistants if needed. The questionnaire collected their demographic information (age, gender, years living in Hong Kong), socioeconomic characteristics (education, household income, occupation, housing type), lifestyle information (alcohol drinking, physical activity), and residential address.

### Personal Exposure Assessment

#### Access to Green Space

At the first visit, a GPS device (BT-Q1000XT, Qstarz International Co Ltd) was distributed to each participant. Research assistants provided the participants with both verbal and paper instructions on device use. The GPS device logged the participant’s location coordinates, date, and time at 1-minute epochs, and the real-time data were automatically collected by a server on the university campus. Raw GPS data were first screened in ArcGIS software (Esri) for missing or suspicious data by comparing them with the daily activity log. Days with less than 600 minutes of GPS data recorded were regarded as missing and excluded from subsequent analysis. The research assistants regularly checked the GPS data collected on the server to ensure the completeness of these data. The participants showed good compliance and yielded no missing data.

We matched cleaned GPS data to a map of the normalized difference vegetation index (NDVI) in the entire territory of Hong Kong to calculate the urban greenness within a 100-, 250-, and 500-meter–radius buffer zone of individual GPS coordinates, based on a Satellite Pour l’Observation de la Terre (SPOT) 6 image obtained in 2016. The NDVI is a normalized ratio of infrared and red bands ranging between –1 and 1, with higher numbers indicating more green vegetation [22]. The NDVI has been widely used in previous environmental studies on the health impacts of green space [23,24]. The average NDVI of individuals was weighted by the daily hours spent in and outside the home.

#### Exposure to Air Pollution and Traffic Noise

We also estimated individual exposure to two ambient air pollutants, nitrogen dioxide (NO_2_) and particulate matter with a diameter less than 2.5 μm (PM_2.5_), by matching the raw GPS coordinates with the hourly maps of PM_2.5_ and NO_2_ at 10-meter spatial resolution. These hourly maps were remodeled from temporal information reported by the Kwai Chung Monitoring Station and adjusted by the spatial patterns of air pollutants reported in previous local studies [25,26]. Weekly average concentrations of NDVI, PM_2.5_, and NO_2_ were calculated from raw data estimated at 1-minute epochs.

The annual average household exposure to road traffic noise was estimated by matching the residential addresses with a 3D-built environment database in Hong Kong that was validated using real-time field data in a local study [27].

#### Physical Activity and Dietary Intake Measurements

At the first visit, an accelerometer data logger (ActiGraph GT3X, ActiGraph LLC) was also distributed to each participant, and the research assistants reminded them to wear the accelerometer on the wrist of their nondominant hand all the time during the study period to continuously record their physical activities. Raw accelerometer data were collected at 1-minute epochs. Individual daily physical activities were calculated from the vector magnitudes of the cleaned data. The cutoff points of light, moderate, and vigorous physical activity were determined according to a formula validated in a Chinese population [28].

We asked each participant to record their daily dietary intakes in weeks 2 and 5 using a standard dietary journal widely used in nutritional studies [29]. Food items and consumed amounts collected from the dietary journals were analyzed with the software Food Processor, version 11.3 (ESHA). The Food Processor database is composed of over 72,000 food items from sources including the United States Department of Agriculture Standard Reference database, Food and Nutrient Database for Dietary Studies, and different manufacturers’ data. Food items, especially local and Chinese food items, which could not be found in the Food Processor database were searched using the Nutrient Information Inquiry System developed by the Centre for Food Safety, the Taiwan Food and Nutrient Database developed by the Food and Drug Administration of Taiwan, or the manufacturers’ webpages. If the nutrition information of the food items still could not be found, the most appropriate food items available were chosen for the analysis. The daily amounts of energy and nutrients consumed, including total calories, calories from fat and saturated fat, protein, carbohydrates, and total fiber, were calculated for individual participants using the Food Processor software.

The above collected data were converted into weekly averages by taking the arithmetic mean—with the exception of the weekly average access to green space, which was calculated using the geometric mean of the NDVI—in the corresponding period between two visits of each participant.

### Outcome Measurements

At each visit, participants first measured their body weight and height, systolic blood pressure, diastolic blood pressure, and heart rate (HR). Lung function tests were conducted using a spirometer (microQuark, COSMED), following the guidelines of the American Thoracic Society [30]. Individual data pertaining to the forced expiratory volume in the first second (FEV1) and forced vital capacity (FVC) were included in the analysis. Each participant donated 5 ml fasting blood at each visit, which was collected in a heparinized tube by a qualified phlebotomist following a standard venipuncture procedure. Specimens were maintained on ice and transported to the laboratory, where blood samples were immediately centrifuged (3000 rpm for 10 minutes at 4 ^o^C) and stored in two 1.0 to 1.5 ml aliquots at –80 ^o^C for batch testing later. The above procedure was completed within 30 minutes of the participant’s arrival at the laboratory to minimize damage to the biomarkers.

We chose a panel of biomarkers used in previous environmental studies on the cardiovascular health effects of green space and air pollution [21,31-34]: (1) metabolic biomarkers: glucose, high-density lipoprotein cholesterol (HDL-C), low-density lipoprotein (LDL-C), total cholesterol (TC), and triglyceride (TG); (2) inflammatory biomarkers: high-sensitivity C-reactive protein (hs-CRP), interleukin-6 (IL-6), tumor necrosis factor α (TNF-α), and soluble platelet selectin (sP-selectin); and 3) oxidative stress biomarkers: 8-hydroxy-2'-deoxyguanosine (8-OHdG), malondialdehyde (MDA), copper-zinc superoxide dismutase (Cu,Zn-SOD), and glutathione peroxidase 1 (GPx-1). The metabolic biomarkers and hs-CRP were measured by an AU480 chemistry analyzer (Beckman Coulter) with corresponding assay kits. The remaining biomarkers were measured using enzyme-linked immunosorbent assay (ELISA) kits purchased from various commercial sources.

### Data Analysis

Spearman coefficients were calculated to assess the correlations between variables. A generalized linear mixed-effects model (GLME), which includes two components, namely, a fixed effect (green space effects and confounding) and a random effect (within-subject variations), was used to estimate the association between green space exposure and biomarker levels or lung functions. The potential confounding factors, including sex, total household income, occupation, physical activities, and household traffic noise exposure, were included as fixed effects in the model. Because there were categorical confounding factors, the generalized version of the variance inflation factor (VIF) was used to detect multicollinearity [35]. A generalized VIF of more than 10 would indicate multicollinearity, and the confounding variables would be removed from the models [36]. Subject numbers were fitted as a random effect variable to account for within-subject variations of repeated measurements.

We fit 4 different models to the weekly measurements of each biomarker to estimate the independent effects of green space. In model 1, the NDVI was added as the only explanatory factor; in model 2, variables of sex, physical activity (moderate to vigorous physical activity levels), occupation, household income, and traffic noise exposure were added to model 1 as confounding factors; in models 3 and 4, PM_2.5_ and NO_2_ were respectively added to model 2 to assess the independent effects of green space exposure with adjustment for ambient air pollutants; and in model 5, daily consumption amounts of protein and carbohydrates were added to model 2 to adjust for the confounding effects of dietary intake. The effects of green space were quantified by the percentage changes of biomarker concentrations associated with a per interquartile range increase of the NDVI. The goodness of fit was evaluated by the Akaike information criterion (AIC), with the minimum indicating the best fit. The likelihood ratio tests of full and partial models were used to show the statistical significance of the variables. All data analysis was conducted using R software, version 3.6.2 (R Foundation for Statistical Computing). The statistical significance was set to *P*<.05. Multiple testing of biomarkers was controlled by the Bonferroni correction.

### Ethical Statement

This project was approved by the Human Subjects Ethics Subcommittee of the Hong Kong Polytechnic University. All participants signed a consent form, and no personal information except residential address was collected in this study.

## Results

### Participant Characteristics

We recruited 40 participants during October to December 2017. One participant withdrew from the study after the first week due to unforeseen family issues. The remaining 39 participants finished all the scheduled visits. The 39 participants had a mean age of 56.4 years (range 50-63 years); 27 (69%) were women, 31 (80%) were married, 35 (90%) were living with family members (family size range 1-6 people), and 14 (36%) received postsecondary education (Table 1).

**Table 1:** Baseline characteristics of the participants (N=39).

Characteristics			Value
Age (years), mean (SD)			56.4 (3.5)
**Sex, n (%)**			
	Male	12 (31)	
	Female	27 (69)	
**Housing type, n (%)**			
	Self-owned	25 (64)	
	Rent	14 (36)	
**Living condition, n (%)**			
	Alone	4 (10)	
	With family member	35 (90)	
Household size, mean (SD)			3.1 (1.2)
**Education level, n (%)**			
	Secondary	25 (64)	
	Postsecondary	14 (36)	
**Occupation, n (%)**			
	Working	21 (54)	
	Homemaker and retired	18 (41)	
**Household income (HK $)^a^, n (%)**			
	Low (10,500 or below)	21 (54)	
	Medium (10,501-23,000)	13 (33)	
	High (23,001 or above)	5 (13)	
**Marital status, n (%)**			
	Married	31 (80)	
	Single	4 (10)	
	Widowed/divorced	4 (10)	
BMI, mean (SD)			23.0 (3.4)

^a^HK $1=US $0.13.

### Outcome Measurements

The weekly average NDVIs at the 100-, 250-, and 500-meter buffer zones were –0.54 (IQR 0.34), –0.61 (IQR 0.25), and –0.61 (IQR 0.25), respectively (Table 2). The weekly average concentrations of PM_2.5_ at the 100-, 250-, and 500-meter buffer zones were 25.57 μg/m^3^ (SD 1.28), 29.26 μg/m^3^ (SD 1.12), and 29.25 μg/m^3^ (SD 1.01). The weekly average concentrations of NO_2_ were 23.46 µg/m^3^ (SD 12.89), 39.73 µg/m^3^ (SD 5.30), and 39.78 μg/m^3^ (SD 5.21) at the 100-, 250-, and 500-meter buffer zones, respectively.

**Table 2:** Weekly average of green space exposure and air pollution for the 3 buffer zones, as well as BMI and physical activity at the 250-meter buffer zone, in the 5-week follow-up period.

Variable			Value, mean (SD)									
			Overall	Week 1		Week 2		Week 3		Week 4		Week 5
**NDVI^a^**												
	100-m buffer zone	–0.54 (0.33)		–0.67 (0.35)	–0.53 (0.34)		–0.53 (0.29)		–0.46 (0.35)		–0.51 (0.33)	
	250-m buffer zone	–0.61 (0.22)		–0.70 (0.29)	–0.61 (0.19)		–0.59 (0.17)		–0.57 (0.21)		–0.61 (0.21)	
	500-m buffer zone	–0.61 (0.22)		–0.70 (0.29)	–0.60 (0.19)		–0.59 (0.17)		–0.57 (0.21)		–0.60 (0.21)	
**PM_2.5_^b^ (µg/m^3^)**												
	100-m buffer zone	25.57 (1.28)		25.60 (1.62)	25.68 (1.20)		25.46 (1.22)		25.67 (1.42)		25.46 (1.07)	
	250-m buffer zone	29.26 (1.12)		29.07 (1.47)	29.25 (1.00)		29.17 (1.05)		29.44 (1.07)		29.35 (1.02)	
	500-m buffer zone	29.25 (1.01)		29.04 (1.44)	29.25 (0.82)		29.18 (0.95)		29.41 (0.89)		29.33 (0.93)	
**NO_2_^c^ (µg/m^3^)**												
	100-m buffer zone	23.46 (12.89)		23.58 (14.84)	23.48 (13.26)		23.63 (12.38)		22.78 (11.82)		23.84 (13.36)	
	250-m buffer zone	39.73 (5.30)		40.43 (8.19)	39.78 (4.00)		39.44 (4.64)		39.08 (4.19)		40.00 (5.20)	
	500-m buffer zone	39.78 (5.21)		40.47 (7.81)	39.83 (4.09)		39.52 (4.56)		39.12 (4.34)		40.03 (5.09)	
BMI (kg/m^2^)			22.83 (3.26)	22.85 (3.30)		22.78 (3.35)		22.89 (3.30)		22.91 (3.29)		22.73 (3.25)
MVPA^d^ (minutes)			177.61 (76.64	180.77 (79.75)		184.02 (79.01)		176.64 (77.24)		172.96 (77.39)		173.91 (73.37)

^a^NDVI: normalized difference vegetation index.

^b^PM_2.5_: particulate matter with a diameter less than 2.5 μm.

^c^NO_2_: nitrogen dioxide.

^d^MVPA: moderate to vigorous physical activity.

Table 3 shows the weekly means and variations of the outcome measures. Overall, these biomarkers show small variations across visits and between individuals, except for the systemic inflammatory biomarkers and some oxidative stress biomarkers. The biomarkers had low to moderate correlations except for the metabolic biomarkers, which had high correlations (Figure S1, Multimedia Appendix 1).

**Table 3:** Summary statistics of the outcome measurements at the weekly visits.

Variable			Outcome, mean (SD)				
		Week 1		Week 2	Week 3	Week 4	Week 5
**Lung functions**							
	FVC^a^ (%)	100.69 (9.65)		99.85 (8.39)	99.67 (10.26)	100.66 (10.06)	98.84 (9.54)
	FEV1^b^ (%)	103.76 (10.43)		102.11 (9.48)	102.01 (11.19)	103.01 (10.62)	101.17 (10.30)
	FEV1/FVC ratio	103.11 (5.02)		102.33 (5.63)	102.37 (5.29)	102.44 (5.90)	102.44 (5.46)
	Heart rate (beats per minute)	71.67 (10.13)		69.46 (9.17)	69.44 (9.25)	71.05 (9.68)	70.28 (9.12)
**Blood pressure (mm Hg)**							
	Systolic	127.64 (15.78)		123.59 (16.28)	124.08 (14.24)	122.77 (16.98)	122.92 (13.02)
	Diastolic	79.90 (12.30)		77.46 (9.60)	77.46 (10.50)	76.18 (10.18)	75.82 (9.07)
**Metabolism biomarkers**							
	Total cholesterol (mmol/L)	5.86 (0.91)		5.72 (1.02)	5.77 (1.07)	5.78 (1.09)	5.52 (1.05)
	Triglycerides (mmol/L)	1.48 (0.82)		1.43 (0.80)	1.44 (0.72)	1.53 (0.86)	1.45 (0.74)
	LDL-C^c^ (mmol/L)	3.83 (0.84)		3.73 (0.92)	3.76 (0.97)	3.73 (0.95)	3.57 (0.92)
	HDL-C^d^ (mmol/L)	1.47 (0.34)		1.44 (0.34)	1.45 (0.33)	1.45 (0.36)	1.38 (0.32)
	Total cholesterol/HDL-C ratio	4.19 (1.12)		4.17 (1.15)	4.16 (1.10)	4.18 (1.10)	4.17 (1.11)
	Triglycerides/HDL-C ratio	1.15 (0.88)		1.14 (0.91)	1.12 (0.76)	1.20 (0.86)	1.18 (0.85)
	LDL-C/HDL-C ratio	2.77 (0.90)		2.75 (0.95)	2.74 (0.92)	2.72 (0.89)	2.72 (0.89)
	Glucose (mmol/L)	5.75 (0.82)		5.60 (0.78)	5.59 (0.72)	5.63 (0.68)	5.45 (0.74)
**Systemic inflammatory biomarkers**							
	hs-CRP^e^ (mg/L)	1.98 (3.11)		1.56 (3.20)	1.01 (0.76)	1.07 (1.20)	1.05 (0.82)
	IL-6^f^ (pg/mL)	1.72 (2.40)		1.78 (2.58)	1.31 (1.66)	1.74 (2.26)	1.25 (1.75)
	TNF-α^g^ (pg/mL)	1.6 (1.22)		1.65 (1.25)	1.49 (1.22)	1.73 (1.45)	1.50 (1.27)
	sP-selectin^h^ (ng/ml)	3.51 (2.62)		3.20 (2.14)	2.90 (1.98)	3.27 (1.89)	3.24 (1.92)
**Oxidative stress biomarkers**							
	MDA^i^ (μM)	40.85 (22.26)		31.83 (21.24)	30.11 (18.88)	36.35 (23.69)	31.75 (19.76)
	8-OHdG^j^ (ng/mL)	1.32 (0.54)		1.41 (0.54)	1.22 (0.39)	1.37 (0.49)	1.47 (0.55)
	SOD^k^ (U/μL)	0.77 (0.38)		0.74 (0.35)	0.77 (0.32)	0.79 (0.36)	0.80 (0.50)
	GPx-1^l^ (ng/mL)	3.25 (7.06)		3.17 (6.91)	2.79 (6.31)	2.54 (5.84)	4.25 (10.8)

^a^FVC: forced vital capacity.

^b^FEV1: forced expiratory volume in the first second.

^c^LDL-C: low-density lipoprotein cholesterol.

^d^HDL-C: high-density lipoprotein cholesterol.

^e^hs-CRP: high-sensitivity C-reactive protein.

^f^IL-6: interleukin-6.

^g^TNF-α: tumor necrosis factor-α.

^h^sP-selectin: soluble platelet selectin.

^i^MDA: malondialdehyde.

^j^8-OHdG: 8-hydroxy-2'-deoxyguanosine.

^k^SOD: superoxide dismutase.

^l^GPx-1: glutathione peroxidase 1.

**Figure 1:**
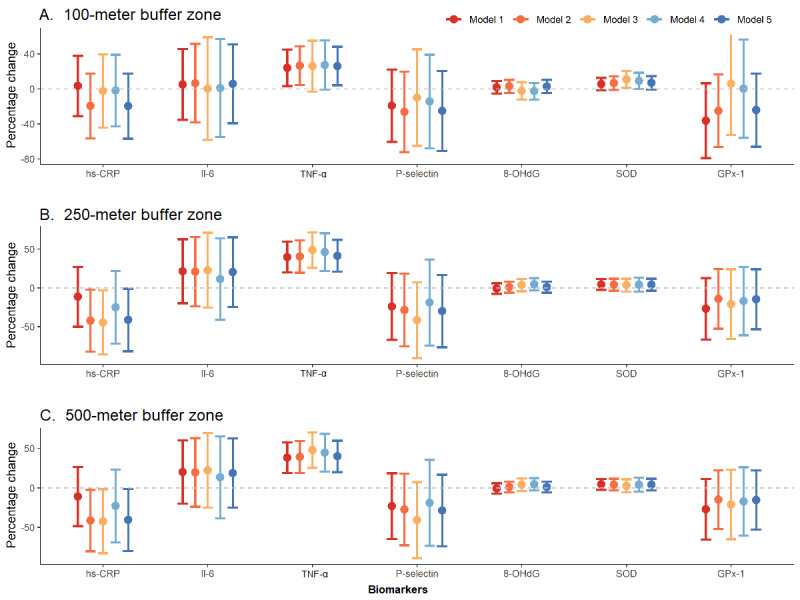
Percentage change in metabolic biomarker concentrations associated with per IQR increase in normalized difference vegetation index (NDVI) at (A) the 100-meter buffer zone, (B) the 250-meter buffer zone, and (C) the 500-meter buffer zone. Vertical bars are 95% CI. Note: model 1: outcome ~ NDVI; model 2: outcome ~ NDVI + covariates (sex, income, occupation, moderate to vigorous physical activity, noise); model 3: outcome ~ NDVI + covariates + PM_2.5_; model 4: outcome ~ NDVI + covariates + NO_2_; model 5: outcome ~ NDVI + covariates + protein + carbohydrates. hs-CRP: high-sensitivity C-reactive protein, IL-6: interleukin-6, TNF-α: tumor necrosis factor α, 8-OHdG: 8-hydroxy-2'-deoxyguanosine, SOD: superoxide dismutase, GPx-1: glutathione peroxidase.

**Figure 2:**
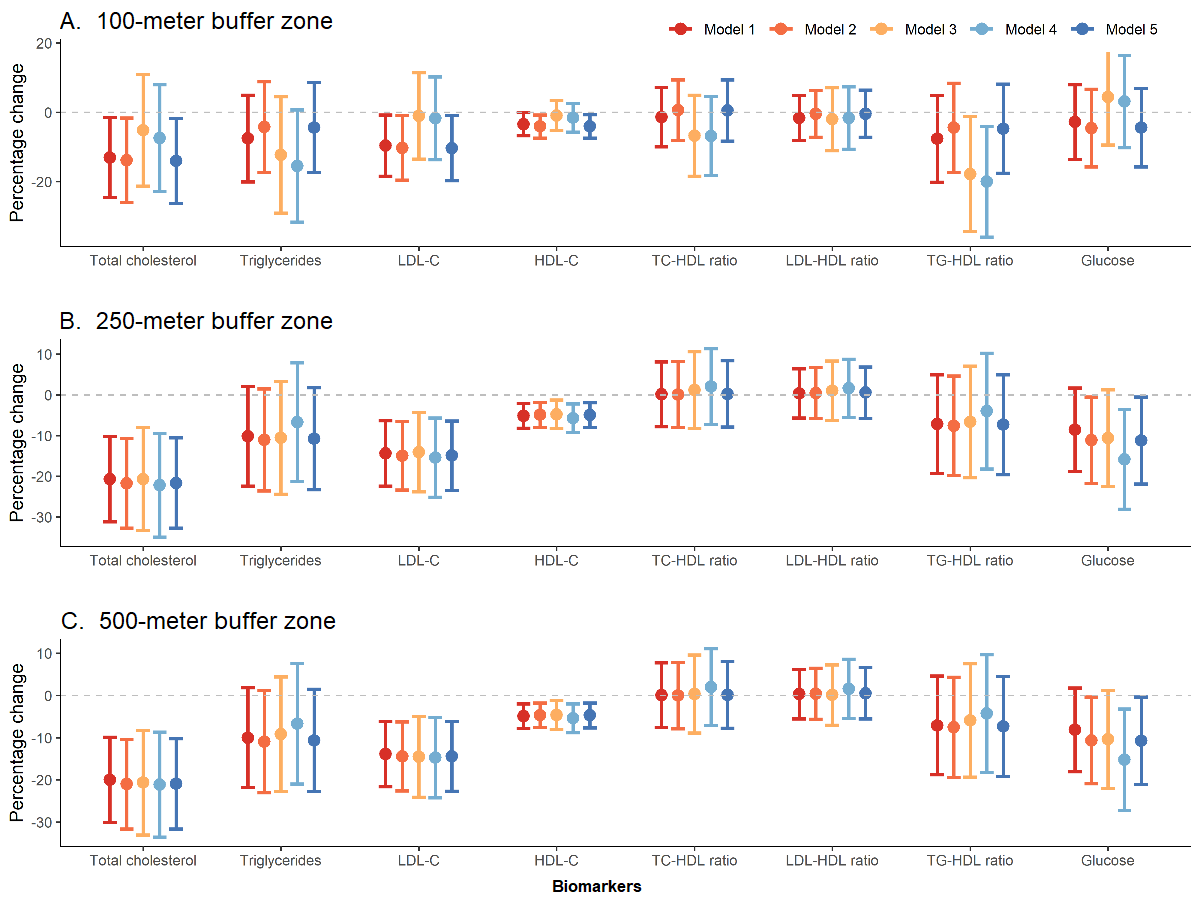
Percentage change in inflammatory and oxidative biomarker concentrations associated with per-IQR increase in normalized difference vegetation index (NDVI) at (A) the 100-meter buffer zone, (B) the 250-meter buffer zone, and (C) the 500-meter buffer zone. Vertical bars are 95% CIs. Note: model 1: outcome ~ NDVI; model 2: outcome ~ NDVI + covariates (sex, income, occupation, moderate to vigorous physical activity, noise); model 3: outcome ~ NDVI + covariates + PM_2.5_; model 4: outcome ~ NDVI + covariates + NO_2_; model 5: outcome ~ NDVI + covariates + protein + carbohydrates. LDL-C: low-density lipoprotein, HDL-C: high-density lipoprotein, TC: total cholesterol, TG: triglyceride.

We compared the AICs of the different GLME models and found that model 3 with the 100-meter NDVI, model 1 with the 250-meter NDVI, and model 1 with the 500-meter NDVI generally returned smaller AICs for most biomarkers than the rest of the models (Table S2, Multimedia Appendix 1). The majority of the generalized VIF values of the variables for all the models was below 2, indicating the absence of collinearity (data not shown). The effect estimates of the NDVI for the 250-meter and 500-meter buffer zones were more consistent between different models, with a few exceptions (TC/HDL ratio and 8-OHdG) (Figures 1 and 2, Tables S3-S5 in Multimedia Appendix 1). Positive associations with NDVI were found for the FEV1/FVC ratio, IL-6, TNF-α, MDA, and SOD, whereas negative associations were found for TC, TG, LDL-C, HDL-C, TG/HDL, LDL/HDL, glucose, and hs-CRP. However, only some estimates reached statistical significance (*P*<.05) at the 250-meter buffer zone, including TC (–21.6% per IQR increase in NDVI, 95% CI –32.7% to –10.6%), LDL-C (–14.9%, 95% CI –23.4% to –6.4%), HDL-C (–4.9%, 95% CI –8.0% to –1.9%), glucose (–11.2%, 95% CI –21.9% to –0.5%), hs-CRP (–41.3%, 95% CI –81.7% to –0.9%), and TNF-α (41.5%, 95% CI 20.9% to 62.0%). The effect estimates of the 100- and 500-meter buffer zones were similar to those of the 250-meter buffer zone except for glucose and hs-CRP, which did not reach statistical significance (*P*>.05) at the 100-meter buffer zone. After adjustment for multiple testing by Bonferroni correction, the estimates remained statistically significant for TC, LDL-C, HDL-C, and TNF-α at the 250- and 500-meter buffer zones (*P*<.002).

## Discussion

### Principal Findings

In this panel study, we found a negative association of NDVI exposure with different metabolic and inflammatory biomarkers. The findings were generally consistent with the beneficial effects of neighborhood green space on biomarkers of respiratory and cardiovascular health in the literature [32], but our study was among the first to use personal tracking devices to objectively and continuously collect individual data of weekly exposure to green space. Different from previous studies using residential greenness as long-term exposure, we adopted a longitudinal study design to explore the effects of weekly exposure to green space on biomarkers of cardiorespiratory health in healthy adults. This reflects subclinical signs for the beneficial or detrimental effects of green space, which could shed light on underlying biological mechanisms that remain unexplored in the literature.

After adjustment for air pollution, physical activities, and dietary intake, we found that lower levels of TC, LDL-C, and fasting glucose were associated with higher green space exposure. Our findings are consistent with 2 cross-sectional studies and 1 cohort study in Chinese populations, which reported that a larger amount of green space in working places or residential areas was associated with lower levels of TC, TG, LDL-C, and fasting glucose [15,37,38]. Our effect estimates are also similar to those reported in a large sample of 15,477 adults in China [37]. However, we found that HDL-C also slightly increased in those with higher green space exposure; this is in contrast to the findings of these two studies, although the LDL/HDL, TG/HDL, and TC/HDL ratios remained unchanged. The simultaneous increase of HDL-C and LDL-C could be due to an intake of high-cholesterol food in some participants, as some previous studies have observed [39]. Hence, the negative associations of all lipid biomarkers may be due to inadequate adjustment for physical activities and dietary intake. Further studies with a larger sample size are needed to elucidate the controversial results of the lipid profile.

Compared to the metabolic biomarkers, the associations of green space and proinflammatory biomarkers were less significant and inconsistent in our study. We found negative associations between green space exposure and hs-CRP, although they were not statistically significant. This echoes the findings of a cohort study of school-aged children in Portugal [40]. Our findings are consistent with a previous study conducted in deprived communities in the United Kingdom that demonstrated an association of residential greenness with hs-CRP [41]. Mao et al [42] conducted an intervention study in 24 older patients with essential hypertension and found that those who stayed in a forest for 7 days and nights had significant reductions in IL-6 but no significant changes in TNF-α. Similar findings were reported in a trial among 20 patients with chronic obstructive pulmonary disease (COPD) after a 1-day forest trip [43]. However, we did not observe any significant effects of green space on IL-6, whereas TNF- levels were consistently higher among those with more green space exposure. The reliability and validity of inflammatory biomarkers to reflect the risk of cardiovascular diseases associated with green space require further study. Other inflammatory biomarkers, such as interleukin-1β, interleukin-8, vascular endothelial growth factor, and soluble tumor necrosis factor-α receptor II [44], could be considered as alternatives in future studies on the health effects of green space.

Elevated MDA and lower SOD levels have been linked to increased risks of coronary artery disease, heart failure, and other chronic diseases [45,46]. It has been proposed that residential green space can reduce oxidative stress and increase angiogenic capacity in patients with cardiovascular diseases [47]. Several experimental studies in patients with hypertension or COPD or in college students found that forest bathing could significantly elevate SOD and lower MDA, despite small sample sizes [48,49]. Our study, however, did not observe significant associations of daily exposure to green space with these oxidative biomarkers. This discrepancy may be due to differences in the study designs and sampling populations.

We did not find any significant effects of green space on lung functions, except FEV1/FVC. This could be due to the fact that the participants were relatively healthy, without any pre-existing chronic respiratory conditions. Sinharay et al [50] conducted a randomized crossover study and found a significant reduction in FEV1 and FVC among patients with COPD, but not among healthy volunteers. Future studies may consider using more sensitive biomarkers for airway inflammation, such as fractional exhaled nitric oxide and differential frequency-dependent respiratory resistance at 5 Hz and 20 Hz, which have used in recent environmental epidemiology studies [50].

Similar to previous green space studies [51,52], we calculated 100-, 250- and 500-meter buffer zones to obtain a comprehensive view of the green space exposure. It is interesting to note that the effect estimates of the 250- and 500-meter buffer zones tend to have consistent patterns but differ from those of the 100-meter buffer zone. By contrast, in a study in Mexican American children in the United States, it was found that children living in residential areas with a higher NDVI had lower odds of dry cough and asthma, and such associations were consistently found across the 3 buffer zones [51]. Other similar studies in children also reported significant associations in the 100-meter buffer zone but not in the larger buffer zones [53]. The daily activity zones of adults are much wider than those of children, which may also explain the lack of significant association of greenness with biomarkers in the 100-meter buffer zone in our adult participants. We speculate that this could also be due to the highly crowded living conditions of Hong Kong, with numerous high-rise residential buildings. Hence, the 250- and 500-meter buffer zones probably better represent community greenness exposure among adults in a metropolitan city such as Hong Kong.

### Limitations

Our study had several limitations. First, the small sample size may render low statistical power, which could explain the few significant effect estimates and wide confidence intervals. Therefore, additional studies with a larger sample size are needed to further elucidate the inconsistent findings. Second, green space exposure was measured by daily average NDVI, which could not reflect the activities performed by participants around the green spaces. Nevertheless, we simultaneously collected the physical activities by personal trackers, which can reduce the confounding effect of these activities. Third, due to a limited budget, we only tested a selected panel of biomarkers, although we attempted to cover a wide range of biomarkers for metabolism, respiratory functions, oxidative stress, and proinflammation. Future studies could adopt more biomarkers to gain a comprehensive understanding of the pathways involved in health effects of green space. Last but not least, sampling bias may exist due to the convenience sampling approach we used in this study. The volunteers tend to be healthier and more educated than the general population, as shown in Table 1. However, the time-invariant characteristics of the participants (such as demographic, lifestyle, and socioeconomic factors) have been well adjusted for in the panel study, because each participant served as their own control. Nevertheless, the weekly green space exposure of these participants had enough variations (as shown in Table 2) to allow us to investigate the effects of green space on different biomarkers. In the future, a large-scale study with a more representative sample of participants could provide more evidence for the health benefits of green space exposure in urban settings.

### Conclusions

By combining data collected via personal tracking devices with green space GIS data, we were able to demonstrate that higher exposure to green space was associated with a better lipid profile and lower inflammatory biomarkers; however, no significant associations were found with respiratory and oxidative biomarkers. The findings of this study provide more clues to the potential biological pathways for the health benefits of green space. From the public health perspective, the health effects of green space identified from this study will also aid the design of future intervention programs to improve the quality of life of the general public.

## Appendix

Multimedia Appendix 1 Supplementary materials.

## Abbreviations

8-OHdG: 8-hydroxy-2'-deoxyguanosine
AIC: Akaike information criterion
COPD: chronic obstructive pulmonary disease
Cu,Zn-SOD: copper–zinc superoxide dismutase
ELISA: enzyme-linked immunosorbent assay
FEV1: forced expiratory volume in the first second
FVC: forced vital capacity
GLME: generalized linear mixed-effects model
GPx-1: glutathione peroxidase
HDL-C: high-density lipoprotein
HR: heart rate
hs-CRP: high-sensitivity C-reactive protein
IHC: Integrative Health Clinic
IL-6: interleukin-6
LDL-C: low-density lipoprotein
MDA: malondialdehyde
NDVI: normalized difference vegetation index
NO_2_: nitrogen dioxide
PM_2.5_: particulate matter with a diameter less than 2.5 μm
sP-selectin: soluble platelet selectin
SPOT: Satellite Pour l’Observation de la Terre
TC: total cholesterol
TG: triglyceride
TNF-α: tumor necrosis factor α
VIF: variance inflation factor
