# Supplementary material for: Effects of Urban Green Space on Cardiovascular and Respiratory Biomarkers in Chinese Adults: Panel Study Using Digital Tracking Devices
Source: JMIR Cardio. 2021 Dec 30;5(2):e31316. doi: 10.2196/31316 (PMC8759022; doi:10.2196/31316)
Supplement: Multimedia Appendix 1 [file cardio_v5i2e31316_app1.docx]

**Multimedia Appendix**

Table S1. Data collection procedure.

|  | 1st Visit  (n=40) | Follow up | | | | |
| --- | --- | --- | --- | --- | --- | --- |
|  |  | Week 1  (n=40) | Week 2  (n=40) | Week 3  (n=39)^a^ | Week 4  (n=39)^a^ | Week 5  (n=39)^a^ |
| Baseline questionnaire | ✔ |  |  |  |  |  |
| Distribute GPS | ✔ |  |  |  |  |  |
| Distribute accelerometer | ✔ |  |  |  |  |  |
| Distribute activity log | ✔ |  |  |  |  |  |
| Distribute dietary journal |  |  | ✔ |  |  | ✔ |
| Measure body weight | ✔ | ✔ | ✔ | ✔ | ✔ | ✔ |
| Measure body height | ✔ | ✔ | ✔ | ✔ | ✔ | ✔ |
| Measure blood pressure | ✔ | ✔ | ✔ | ✔ | ✔ | ✔ |
| Measure heart rate | ✔ | ✔ | ✔ | ✔ | ✔ | ✔ |
| Spirometer | ✔ | ✔ | ✔ | ✔ | ✔ | ✔ |
| Blood drawing | ✔ | ✔ | ✔ | ✔ | ✔ | ✔ |
| Collect GPS data |  | ✔ | ✔ | ✔ | ✔ | ✔ |
| Collect accelerometer data |  | ✔ | ✔ | ✔ | ✔ | ✔ |
| Collect activity log |  | ✔ | ✔ | ✔ | ✔ | ✔ |
| Deliver Coupon | ✔ | ✔ | ✔ | ✔ | ✔ | ✔ |
| Collect GPS device |  |  |  |  |  | ✔ |
| Collect accelerometer device |  |  |  |  |  | ✔ |

^a^One participant withdrew from the study.

Figure S1. Correlation matrix between variables.


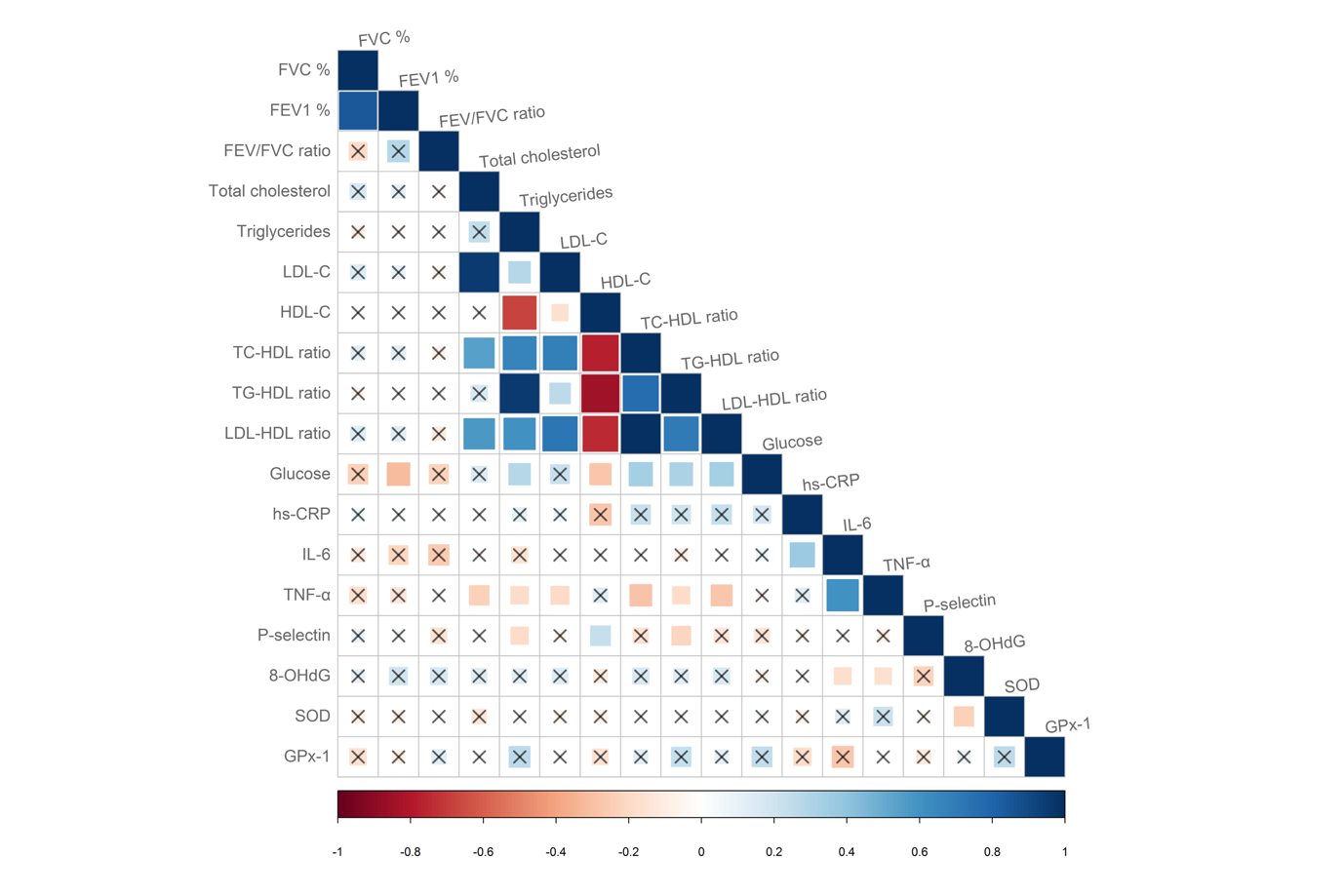


Table S2. Comparison of the goodness-of-fit between different models.

|  | **100m NDVI** | | | | | **250m NDVI** | | | | | **500m NDVI** | | | | |
| --- | --- | --- | --- | --- | --- | --- | --- | --- | --- | --- | --- | --- | --- | --- | --- |
| **AIC** | **Model 1** | **Model 2** | **Model 3** | **Model 4** | **Model 5** | **Model 1** | **Model 2** | **Model 3** | **Model 4** | **Model 5** | **Model 1** | **Model 2** | **Model 3** | **Model 4** | **Model 5** |
| FVC % | 1019.1 | 989.9 | 919.2 | 927.2 | 1006.6 | 1018.7 | 989.5 | 990.6 | 992.7 | 1002.4 | 1018.7 | 986.7 | 989.4 | 987.8 | 990.1 |
| FEV1 % | 1029.5 | 997.5 | 914.7 | 924.7 | 1013.9 | 1029.1 | 997.0 | 998.3 | 998.9 | 1010.0 | 1029.1 | 994.5 | 996.9 | 995.4 | 996.3 |
| FEV1/FVC ratio | 841.7 | 823.5 | 753.5 | 762.2 | 841.2 | 841.0 | 822.9 | 824.9 | 828.1 | 837.1 | 841.2 | 819.3 | 823.1 | 821.5 | 823.9 |
| Total Cholesterol | 266.3 | 290.8 | 273.2 | 277.2 | 316.5 | 256.5 | 281.1 | 286.8 | 290.1 | 298.8 | 256.6 | 274.0 | 281.1 | 279.7 | 282.9 |
| Triglycerides | 286.6 | 301.3 | 280.9 | 287.7 | 323.4 | 284.8 | 298.1 | 303.8 | 305.6 | 314.3 | 284.7 | 290.3 | 298.0 | 295.4 | 297.9 |
| LDL-C | 183.6 | 212.3 | 204.5 | 207.7 | 239.3 | 175.8 | 204.9 | 211.2 | 214.4 | 223.1 | 175.9 | 197.9 | 205.0 | 204.0 | 207.2 |
| HDL-C | -149.6 | -121.3 | -107.3 | -104.2 | -90.4 | -156.6 | -125.5 | -117.1 | -114.8 | -105.7 | -156.4 | -129.7 | -125.3 | -121.5 | -118.7 |
| TC/HDL ratio | 184.7 | 202.3 | 194.1 | 201.3 | 228.8 | 184.4 | 201.9 | 208.1 | 210.8 | 220.1 | 184.4 | 196.5 | 201.9 | 202.7 | 205.4 |
| TG/HDL ratio | 285.0 | 296.6 | 276.4 | 282.0 | 320.1 | 284.6 | 295.1 | 300.7 | 303.0 | 311.8 | 284.5 | 287.5 | 295.0 | 292.8 | 295.6 |
| LDL/HDL ratio | 97.2 | 123.5 | 124.9 | 132.2 | 151.4 | 97.0 | 123.1 | 129.9 | 132.8 | 141.8 | 97.0 | 117.7 | 123.1 | 124.4 | 127.4 |
| Glucose | 234.5 | 253.8 | 226.4 | 231.0 | 279.6 | 231.7 | 249.8 | 255.8 | 256.8 | 267.6 | 231.8 | 242.1 | 249.9 | 247.9 | 249.3 |
| hs-CRP | 731.3 | 707.2 | 655.4 | 663.0 | 727.9 | 730.1 | 703.4 | 707.0 | 708.3 | 718.9 | 730.1 | 699.3 | 703.3 | 702.7 | 702.8 |
| IL-6 | 699.1 | 698.8 | 659.3 | 665.9 | 719.2 | 697.5 | 697.4 | 700.7 | 703.2 | 711.6 | 697.6 | 691.8 | 697.5 | 694.9 | 697.9 |
| TNF-α | 458.9 | 468.7 | 440.2 | 449.0 | 488.6 | 448.7 | 459.7 | 461.8 | 466.7 | 468.7 | 448.9 | 455.5 | 459.8 | 457.9 | 462.1 |
| P-selectin | 720.2 | 715.7 | 662.4 | 670.1 | 734.8 | 719.2 | 714.9 | 716.0 | 720.8 | 728.8 | 719.2 | 710.0 | 715.0 | 710.6 | 715.6 |
| MDA | 1514.9 | 1455.5 | 1340.6 | 1352.6 | 1467.0 | 1515.3 | 1457.3 | 1456.2 | 1458.6 | 1467.3 | 1515.3 | 1455.8 | 1457.3 | 1454.2 | 1456.9 |
| 8-OHdG | 94.1 | 125.2 | 127.2 | 131.9 | 152.3 | 93.7 | 125.2 | 130.0 | 132.0 | 143.0 | 93.8 | 117.1 | 125.2 | 120.8 | 124.2 |
| SOD | 104.3 | 139.7 | 143.4 | 148.8 | 166.5 | 104.3 | 140.7 | 147.5 | 150.5 | 159.1 | 104.3 | 132.4 | 140.7 | 138.6 | 142.1 |
| GPx-1 | 741.1 | 711.5 | 671.0 | 676.5 | 732.1 | 741.7 | 711.9 | 714.9 | 718.4 | 726.8 | 741.5 | 708.5 | 711.9 | 711.3 | 714.8 |
| HR | 1137.2 | 1099.6 | 1009.7 | 1018.7 | 1112.2 | 1137.6 | 1099.9 | 1100.3 | 1101.4 | 1108.7 | 1137.7 | 1097.1 | 1099.9 | 1097.3 | 1098.8 |
| SBP | 1274.5 | 1226.9 | 1137.1 | 1148.3 | 1239.0 | 1275.6 | 1227.3 | 1227.1 | 1228.6 | 1238.0 | 1275.6 | 1229.5 | 1227.3 | 1228.8 | 1230.5 |
| DBP | 1185.1 | 1139.0 | 1016.2 | 1032.4 | 1153.3 | 1183.9 | 1138.0 | 1138.5 | 1140.2 | 1148.8 | 1183.7 | 1137.7 | 1137.8 | 1137.8 | 1140.1 |

Table S3. Effect estimates associated with per IQR increase in NDVI at the 100m buffer zone.

|  | **Model 1** |  | **Model 2** |  | **Model 3** |  | **Model 4** |  | **Model 5** |  |
| --- | --- | --- | --- | --- | --- | --- | --- | --- | --- | --- |
| **Outcomes** | **Percentage change**  **(95% CI)** | ***P*** | **Percentage change**  **(95% CI)** | ***P*** | **Percentage change**  **(95% CI)** | ***P*** | **Percentage change**  **(95% CI)** | ***P*** | **Percentage change**  **(95% CI)** | ***P*** |
| FVC % | -62.7 (-169.9, 44.5) | .254 | -85.9 (-198, 26.3) | .136 | 34.1 (-119.4, 187.6) | .664 | 18.4 (-128.4, 165.2) | .807 | -83.8 (-196.6, 28.9) | .147 |
| FEV1 % | -37.1 (-146.8, 72.6) | .509 | -47.5 (-161.9, 66.9) | .417 | 132.7 (-17.1, 282.6) | .085 | 104.7 (-40, 249.4) | .158 | -46.5 (-161.5, 68.5) | .429 |
| FEV1/FVC ratio | 30.8 (-33.2, 94.8) | .347 | 42.6 (-23.7, 108.8) | .210 | 101.1 (15.3, 187) | .022 | 88.7 (6, 171.4) | .037 | 41.5 (-24.9, 107.9) | .223 |
| Sleep efficiency | 8.1 (-57, 73.3) | .807 | 23.3 (-41, 87.6) | .478 | -29.1 (-109.5, 51.3) | .480 | -36.1 (-113.1, 41) | .360 | 24 (-40.7, 88.7) | .469 |
| Total Cholesterol | -13.0 (-24.6, -1.5) | .028 | -13.8 (-26, -1.6) | .028 | -5.1 (-21.3, 11) | .533 | -7.4 (-22.9, 8) | .348 | -14 (-26.3, -1.7) | .027 |
| Triglycerides | -7.6 (-20.1, 5) | .240 | -4.2 (-17.3, 8.8) | .527 | -12.3 (-29.1, 4.5) | .153 | -15.5 (-31.7, 0.7) | .063 | -4.4 (-17.4, 8.6) | .509 |
| LDL-C | -9.6 (-18.5, -0.8) | .034 | -10.3 (-19.6, -1) | .032 | -1 (-13.5, 11.4) | .872 | -1.7 (-13.7, 10.3) | .782 | -10.4 (-19.7, -1) | .032 |
| HDL-C | -3.4 (-6.8, -0.1) | .045 | -4.1 (-7.4, -0.8) | .017 | -1 (-5.3, 3.4) | .665 | -1.6 (-5.7, 2.6) | .465 | -4 (-7.4, -0.7) | .019 |
| TC/HDL ratio | -1.4 (-10, 7.2) | .753 | 0.6 (-8.2, 9.4) | .893 | -6.7 (-18.4, 5) | .262 | -6.8 (-18.3, 4.6) | .246 | 0.5 (-8.3, 9.3) | .904 |
| TG/HDL ratio | -7.6 (-20.1, 4.9) | .234 | -4.4 (-17.3, 8.4) | .500 | -17.8 (-34.4, -1.2) | .037 | -20 (-36, -4.1) | .015 | -4.7 (-17.6, 8.1) | .474 |
| LDL/HDL ratio | -1.6 (-8.1, 4.9) | .629 | -0.4 (-7.1, 6.4) | .908 | -2 (-11.1, 7.1) | .670 | -1.6 (-10.6, 7.4) | .724 | -0.4 (-7.2, 6.4) | .910 |
| Glucose | -2.8 (-13.5, 8) | .613 | -4.5 (-15.8, 6.7) | .429 | 4.5 (-9.5, 18.4) | .530 | 3.2 (-10.2, 16.5) | .644 | -4.4 (-15.7, 6.9) | .445 |
| hs-CRP | 3.4 (-31.1, 37.8) | .849 | -19.5 (-56.6, 17.6) | .306 | -2.5 (-44.3, 39.4) | .909 | -1.8 (-42.7, 39.1) | .932 | -19.6 (-57, 17.7) | .307 |
| IL-6 | 5 (-35.5, 45.5) | .809 | 6.5 (-38.5, 51.5) | .777 | 0.3 (-58.4, 59.1) | .991 | 1 (-55.2, 57.2) | .971 | 5.7 (-39.4, 50.9) | .803 |
| TNF-α | 24.1 (3.3, 45.0) | .025 | 26.6 (4.4, 48.7) | .020 | 26 (-3, 55.1) | .081 | 27.3 (-0.8, 55.4) | .059 | 26.2 (4.1, 48.3) | .022 |
| P-selectin | -19.3 (-60.6, 22.1) | .363 | -26.2 (-72.2, 19.7) | .265 | -10 (-65.3, 45.4) | .725 | -14.3 (-67.8, 39.2) | .602 | -25.2 (-71, 20.6) | .284 |
| MDA | 246.5 (-141.6, 634.5) | .217 | 366.2 (-65.4, 797.8) | .100 | 548.1 (23.6, 1072.7) | .045 | 501.1 (-2.1, 1004.3) | .055 | 368.9 (-67.7, 805.6) | .101 |
| 8-OHdG | 1.8 (-5.3, 8.9) | .627 | 3 (-4.5, 10.4) | .437 | -2.2 (-12.1, 7.7) | .663 | -2.6 (-12.1, 6.9) | .592 | 2.8 (-4.7, 10.3) | .463 |
| SOD | 5.4 (-1.7, 12.5) | .136 | 6.7 (-1, 14.3) | .092 | 10.8 (1.2, 20.4) | .029 | 9.2 (0, 18.5) | .053 | 6.9 (-0.8, 14.6) | .082 |
| GPx-1 | -36.3 (-79, 6.4) | .098 | -25.1 (-66.6, 16.5) | .239 | 6 (-52.6, 64.6) | .842 | 0.2 (-56, 56.5) | .994 | -24.3 (-66.1, 17.6) | .258 |
| HR | 83.9 (-71.4, 239.2) | .291 | 79.9 (-83.5, 243.2) | .339 | 223.1 (14, 432.2) | .038 | 194.2 (-5.5, 393.8) | .059 | 72.8 (-90.2, 235.9) | .383 |
| SBP | -157.8 (-391.5, 75.9) | .188 | -131.4 (-375.7, 112.9) | .293 | -109.9 (-436.1, 216.4) | .510 | -149.6 (-463.1, 164) | .351 | -126.8 (-371.6, 118.1) | .312 |
| DBP | 86.9 (-91.3, 265) | .341 | 152.5 (-31.7, 336.7) | .107 | 108.3 (-103.5, 320.2) | .318 | 57.2 (-149.8, 264.3) | .589 | 146.1 (-38.7, 331) | .123 |

Note: CI, confidence interval. Model 1: Outcome ~ NDVI ; Model 2: Outcome ~ NDVI + Covariates (sex, income, occupation, MVPA, noise) ; Model 3: Outcome ~ NDVI + Covariates + PM_2.5_; Model 4: Outcome ~ NDVI + Covariates + NO_2_; Model 5: Outcome ~ NDVI + Covariates + protein + carbohydrate

Table S4. Effect estimates associated with per IQR increase in NDVI at the 250m buffer zone.

|  | **Model 1** |  | **Model 2** |  | **Model 3** |  | **Model 4** |  | **Model 5** |  |
| --- | --- | --- | --- | --- | --- | --- | --- | --- | --- | --- |
| **Outcomes** | **Percentage change 95% CI** | ***P*** | **Percentage change 95% CI** | ***P*** | **Percentage change 95% CI** | ***P*** | **Percentage change 95% CI** | ***P*** | **Percentage change 95% CI** | ***P*** |
| FVC % | -55.3 (-156.4, 45.8) | .285 | -80.2 (-185.4, 25.1) | .138 | -94.4 (-214.7, 26) | .127 | -115.5 (-237, 6) | .065 | -81.3 (-187, 24.3) | .134 |
| FEV1 % | -28.6 (-131.7, 74.5) | .588 | -43.3 (-150.5, 63.9) | .430 | -45.7 (-168.7, 77.2) | .467 | -93.4 (-216.3, 29.5) | .139 | -45.4 (-153, 62.2) | .410 |
| FEV1/FVC ratio | 31.7 (-28.7, 92.2) | .305 | 40.5 (-21.7, 102.7) | .204 | 52.4 (-18.7, 123.6) | .151 | 28.7 (-43, 100.3) | .435 | 39.5 (-22.8, 101.8) | .216 |
| Total Cholesterol | -20.7 (-31.2, -10.3) | <.001 | -21.7 (-32.7, -10.7) | <.001 | -20.7 (-33.3, -8.1) | .002 | -22.2 (-34.9, -9.5) | .001 | -21.6 (-32.7, -10.6) | <.001 |
| Triglycerides | -10.2 (-22.4, 2) | .104 | -11.1 (-23.6, 1.4) | .085 | -10.6 (-24.4, 3.3) | .137 | -6.7 (-21.3, 7.8) | .367 | -10.8 (-23.3, 1.7) | .093 |
| LDL-C | -14.4 (-22.4, -6.4) | .001 | -14.9 (-23.4, -6.5) | .001 | -14.1 (-23.8, -4.3) | .005 | -15.4 (-25.1, -5.7) | .002 | -14.9 (-23.4, -6.4) | .001 |
| HDL-C | -5.2 (-8.2, -2.1) | .001 | -4.9 (-7.9, -1.9) | .002 | -4.8 (-8.3, -1.3) | .008 | -5.7 (-9.2, -2.2) | .002 | -4.9 (-8, -1.9) | .002 |
| TC/HDL ratio | 0.1 (-7.8, 8.1) | .976 | 0.1 (-8.1, 8.2) | .988 | 1.1 (-8.3, 10.6) | .814 | 2.1 (-7.3, 11.4) | .666 | 0.2 (-7.9, 8.4) | .955 |
| TG/HDL ratio | -7.2 (-19.3, 4.9) | .247 | -7.6 (-19.8, 4.7) | .227 | -6.7 (-20.4, 7) | .342 | -4 (-18.2, 10.2) | .582 | -7.3 (-19.6, 5) | .244 |
| LDL/HDL ratio | 0.3 (-5.7, 6.4) | .912 | 0.5 (-5.8, 6.7) | .886 | 1 (-6.3, 8.3) | .786 | 1.6 (-5.6, 8.8) | .663 | 0.5 (-5.7, 6.8) | .864 |
| Glucose | -8.5 (-18.8, 1.7) | .104 | -11.1 (-21.7, -0.5) | .042 | -10.6 (-22.5, 1.3) | .082 | -15.9 (-28.1, -3.6) | .012 | -11.2 (-21.9, -0.5) | .042 |
| hs-CRP | -11.4 (-50.1, 27.2) | .564 | -42.3 (-82.2, -2.3) | .041 | -44.7 (-86, -3.4) | .037 | -25 (-72, 22) | .300 | -41.3 (-81.7, -0.9) | .048 |
| IL-6 | 21.5 (-19.8, 62.8) | .310 | 21.1 (-23.7, 65.9) | .358 | 22.8 (-25.5, 71.2) | .356 | 11.4 (-41.2, 64.1) | .671 | 20.3 (-24.7, 65.4) | .377 |
| TNF-α | 39.7 (19.9, 59.6) | <.001 | 40.6 (19.7, 61.4) | <.001 | 48.7 (25.8, 71.6) | <.001 | 46.1 (21.9, 70.4) | <.001 | 41.5 (20.9, 62) | <.001 |
| P-selectin | -23.9 (-67, 19.2) | .278 | -28.5 (-75.3, 18.2) | .234 | -41.6 (-90.4, 7.3) | .098 | -18.7 (-74.2, 36.7) | .509 | -29.9 (-76.6, 16.7) | .211 |
| MDA | 127.3 (-296.8, 551.3) | .558 | 90.4 (-377.3, 558.1) | .706 | 109.9 (-377.5, 597.3) | .659 | 194.1 (-364.1, 752.3) | .497 | 95.8 (-376.9, 568.5) | .692 |
| 8-OHdG | -0.8 (-7.6, 6) | .812 | 0.9 (-6.1, 8) | .793 | 3.6 (-4.3, 11.5) | .375 | 4.7 (-3.4, 12.7) | .260 | 1.1 (-6, 8.2) | .769 |
| SOD | 4.6 (-2.6, 11.8) | .217 | 4.2 (-3.5, 11.8) | .291 | 3.6 (-4.8, 11.9) | .403 | 4.2 (-4.9, 13.2) | .368 | 4.1 (-3.6, 11.8) | .297 |
| GPx-1 | -26.9 (-66.5, 12.7) | .186 | -14.2 (-52.6, 24.3) | .471 | -20.8 (-65.9, 24.2) | .366 | -17 (-61.2, 27.1) | .451 | -14.7 (-53.4, 23.9) | .456 |
| HR | 30.3 (-122.2, 182.7) | .698 | 20.9 (-137.1, 178.9) | .796 | 41.3 (-133.8, 216.5) | .644 | -50.2 (-232.6, 132.3) | .591 | 21.8 (-135.8, 179.4) | .787 |
| SBP | 22.0 (-205.4, 249.5) | .850 | 41.9 (-194, 277.7) | .728 | 25.3 (-236.8, 287.5) | .850 | -48.3 (-321.5, 225) | .730 | 37.1 (-198.8, 273) | .758 |
| DBP | 111.2 (-64.1, 286.4) | .216 | 159.5 (-20.6, 339.7) | .084 | 165.7 (-31.3, 362.7) | .101 | 97.6 (-112.5, 307.7) | .364 | 159.4 (-21.5, 340.2) | .086 |

Note: CI, confidence interval. Model 1: Outcome ~ NDVI ; Model 2: Outcome ~ NDVI + Covariates (sex, income, occupation, MVPA, noise) ; Model 3: Outcome ~ NDVI + Covariates + PM_2.5_; Model 4: Outcome ~ NDVI + Covariates + NO_2_; Model 5: Outcome ~ NDVI + Covariates + protein + carbohydrate

Table S5. Effect estimates associated with per IQR increase in NDVI at the 500m buffer zone.

|  | **Model 1** |  | **Model 2** |  | **Model 3** |  | **Model 4** |  | **Model 5** |  |
| --- | --- | --- | --- | --- | --- | --- | --- | --- | --- | --- |
| **Outcomes** | **Percentage change**  **(95% CI)** | ***P*** | **Percentage change**  **(95% CI)** | ***P*** | **Percentage change**  **(95% CI)** | ***P*** | **Percentage change**  **(95% CI)** | ***P*** | **Percentage change**  **(95% CI)** | ***P*** |
| FVC % | -54.2 (-151.9, 43.5) | .279 | -78.7 (-180.5, 23.2) | .132 | -89.4 (-207.4, 28.6) | .140 | -108.8 (-228, 10.4) | .076 | -79.6 (-181.9, 22.6) | .129 |
| FEV1 % | -30 (-129.6, 69.7) | .556 | -44.7 (-148.4, 59) | .399 | -58.4 (-178.6, 61.9) | .343 | -94 (-214.4, 26.5) | .129 | -46.8 (-150.8, 57.3) | .380 |
| FEV1/FVC ratio | 28.8 (-29.7, 87.3) | .336 | 37.2 (-23, 97.4) | .228 | 35 (-34.7, 104.8) | .327 | 20.8 (-49.4, 90.9) | .563 | 36 (-24.3, 96.3) | .243 |
| Total Cholesterol | -20.0 (-30.1, -9.9) | <.001 | -21 (-31.6, -10.4) | <.001 | -20.6 (-33, -8.3) | .001 | -21.1 (-33.5, -8.7) | .001 | -20.9 (-31.7, -10.2) | <.001 |
| Triglycerides | -10.0 (-21.8, 1.8) | .099 | -10.9 (-23.1, 1.2) | .079 | -9.2 (-22.7, 4.4) | .188 | -6.7 (-21, 7.6) | .362 | -10.6 (-22.7, 1.5) | .087 |
| LDL-C | -13.9 (-21.6, -6.1) | .001 | -14.4 (-22.6, -6.3) | .001 | -14.5 (-24.1, -5) | .003 | -14.7 (-24.2, -5.2) | .003 | -14.4 (-22.6, -6.2) | .001 |
| HDL-C | -4.9 (-7.9, -2) | .001 | -4.7 (-7.6, -1.7) | .002 | -4.6 (-8.1, -1.2) | .009 | -5.4 (-8.9, -2) | .002 | -4.7 (-7.7, -1.8) | .002 |
| TC/HDL ratio | 0.1 (-7.6, 7.8) | .988 | 0 (-7.9, 7.8) | .996 | 0.3 (-8.9, 9.6) | .945 | 2 (-7.1, 11.1) | .670 | 0.2 (-7.7, 8) | .970 |
| TG/HDL ratio | -7.1 (-18.8, 4.6) | .237 | -7.5 (-19.4, 4.3) | .214 | -5.9 (-19.3, 7.5) | .390 | -4.2 (-18.2, 9.7) | .552 | -7.3 (-19.2, 4.6) | .230 |
| LDL/HDL ratio | 0.3 (-5.5, 6.2) | .914 | 0.4 (-5.6, 6.5) | .891 | 0.1 (-7, 7.2) | .975 | 1.5 (-5.5, 8.6) | .667 | 0.5 (-5.6, 6.6) | .867 |
| Glucose | -8.1 (-18, 1.8) | .110 | -10.6 (-20.9, -0.4) | .044 | -10.4 (-22.1, 1.2) | .082 | -15.2 (-27.2, -3.2) | .014 | -10.7 (-21.1, -0.4) | .044 |
| hs-CRP | -11 (-48.4, 26.4) | .566 | -41.4 (-80.2, -2.6) | .039 | -42.4 (-82.9, -1.9) | .043 | -22.9 (-69, 23.2) | .333 | -40.6 (-79.8, -1.4) | .045 |
| IL-6 | 19.9 (-20, 59.9) | .330 | 19.6 (-23.8, 62.9) | .378 | 22.2 (-25.2, 69.7) | .360 | 13.3 (-38.5, 65) | .616 | 18.8 (-24.8, 62.4) | .400 |
| TNF-α | 38.2 (18.9, 57.4) | <.001 | 39.1 (18.9, 59.3) | <.001 | 47.7 (25.2, 70.2) | <.001 | 44.6 (20.8, 68.4) | <.001 | 39.9 (20, 59.8) | <.001 |
| P-selectin | -23.1 (-64.8, 18.5) | .278 | -27.4 (-72.8, 17.9) | .238 | -40.7 (-88.8, 7.3) | .099 | -19 (-73.4, 35.4) | .494 | -28.6 (-73.9, 16.6) | .217 |
| MDA | 125.3 (-284.6, 535.2) | .550 | 89.1 (-364.8, 543) | .701 | 127.8 (-350.3, 605.9) | .601 | 229 (-318.4, 776.4) | .414 | 93.9 (-364.7, 552.4) | .689 |
| 8-OHdG | -0.8 (-7.4, 5.8) | .808 | 0.9 (-5.9, 7.7) | .795 | 4 (-3.7, 11.8) | .310 | 4.5 (-3.4, 12.5) | .264 | 1 (-5.9, 7.9) | .773 |
| SOD | 4.4 (-2.5, 11.4) | .213 | 4.1 (-3.4, 11.5) | .286 | 2.6 (-5.5, 10.8) | .530 | 4.1 (-4.8, 13) | .366 | 4.1 (-3.4, 11.5) | .290 |
| GPx-1 | -27.1 (-65.3, 11.2) | .168 | -14.9 (-52, 22.3) | .435 | -21.1 (-65.1, 22.9) | .350 | -17.1 (-60.4, 26.1) | .439 | -15.4 (-52.8, 22) | .422 |
| cortisol | -170.2 (-1804.6, 1464.1) | .838 | -252.5 (-1996.7, 1491.8) | .777 | -55.1 (-1940.2, 1830) | .954 | 103 (-1982.6, 2188.5) | .923 | -248.9 (-2000.6, 1502.8) | .781 |
| Heart rate | 27.3 (-120.2, 174.7) | .718 | 18.2 (-134.7, 171.1) | .816 | 40.3 (-131.5, 212) | .646 | -51.9 (-230.8, 127) | .570 | 18.3 (-134.3, 170.9) | .814 |
| SBP | 15 (-204.9, 234.9) | .894 | 33.4 (-194.9, 261.7) | .775 | -9.8 (-266.6, 246.9) | .940 | -48.6 (-316.9, 219.8) | .723 | 28.9 (-199.5, 257.3) | .805 |
| DBP | 112.9 (-56.4, 282.3) | .193 | 160.1 (-14.2, 334.3) | .074 | 171.7 (-21.4, 364.8) | .083 | 115.9 (-90.5, 322.2) | .273 | 159.4 (-15.6, 334.4) | .076 |

Note: CI, confidence interval. Model 1: Outcome ~ NDVI ; Model 2: Outcome ~ NDVI + Covariates (sex, income, occupation, MVPA, noise) ; Model 3: Outcome ~ NDVI + Covariates + PM_2.5_; Model 4: Outcome ~ NDVI + Covariates + NO_2_; Model 5: Outcome ~ NDVI + Covariates + protein + carbohydrate
